# Communicating medication risks in pregnancy: Towards shared decision making

**DOI:** 10.3389/fdsfr.2025.1712216

**Published:** 2025-10-16

**Authors:** Ursula Winterfeld, Kenneth Hodson, Maya Berlin, Benoît Marin, Michael Ceulemans, Corinna Weber-Schoendorfer, François R. Girardin, David Baud, Béatrice Schaad, Alice Panchaud

**Affiliations:** ^1^ Swiss Teratogen Information Service and Clinical Pharmacology Service, Lausanne University Hospital, Lausanne, Switzerland; ^2^ UK Teratology Information Service, Newcastle uponTyne, United Kingdom; ^3^ Directorate of Women’s Services, Royal Victoria Infirmary, Newcastle uponTyne, United Kingdom; ^4^ Clinical Pharmacology and Toxicology Unit, Drug Consultation Center, Shamir Medical Center affiliated with Faculty of Medical & Health Science, Tel Aviv University, Tel Aviv, Israel; ^5^ Département de Santé Publique, Sorbonne Université, INSERM, Institut Pierre Louis d’Epidémiologie et de Santé Publique, AP-HP, Hôpital Trousseau, Centre de Référence sur les Agents Tératogènes (CRAT), Paris, France; ^6^ Department of Pharmaceutical and Pharmacological Sciences, Leuven, Belgium; ^7^ KU Leuven Child & Youth Institute, Leuven, Belgium; ^8^ IQ Health, Radboud University Medical Center, Nijmegen, Netherlands; ^9^ Charité – Universitätsmedizin Berlin, Corporate Member of Freie Universität Berlin, Humboldt-Universität zu Berlin, and Berlin Institute of Health, Pharmakovigilanzzentrum Embryonaltoxikologie, Institut für Klinische Pharmakologie und Toxikologie, Berlin, Germany; ^10^ Service of Clinical Pharmacology, Department of Medicine, Lausanne University Hospital and University of Lausanne, Lausanne, Switzerland; ^11^ Materno-Fetal and Obstetrics Research Unit, Department “Woman-Mother-Child”, Lausanne University Hospital, Lausanne, Switzerland; ^12^ Communication Department, Lausanne University Hospital, Lausanne, Switzerland; ^13^ Institute of Humanities in Medicine, Lausanne University Hospital and University of Lausanne, Lausanne, Switzerland; ^14^ Service of Pharmacy, Lausanne University Hospital and University of Lausanne, Lausanne, Switzerland; ^15^ Institute of Primary Healthcare (BIHAM), University of Bern, Bern, Switzerland

**Keywords:** medication, pregnancy, risk communication, shared decision making, pharmacovgilance

## Abstract

Medication use during pregnancy is common, yet safety data remain limited, often leading to exaggerated risk perceptions and suboptimal care. Current communication practices are fragmented: product labeling and patient leaflets tend to emphasize potential harms, while specialized resources are underused. We explore the challenges of conveying accurate risk-benefit information and highlight the need for shared decision making. Integrated, evidence-based resources accessible to both healthcare providers and patients can reduce misinformation, improve adherence to essential treatments, and ultimately enhance maternal and child health. The Swiss MAMA-MEDS initiative illustrates how a unified knowledge base can align messaging, address health inequalities, and support patient-centered pregnancy care.

## 1 Introduction

In 1961, thalidomide as an unforeseen human teratogenic drug led to the exclusion of most women of childbearing potential and pregnant women from clinical trials (Gerbier and Panchaud, 2023). Consequently, our knowledge about drug safety in pregnancy is reliant on observational data collected post-marketing, with a significant time lag between a drug’s initial approval and the availability of essential pharmacokinetic and safety data specific to pregnancy (Gerbier and Panchaud, 2023). The result is highly restrictive drug labelling, often limited to “do not use during pregnancy” or “use with caution during pregnancy” recommendations. Extensive and supportive knowledge about a drug is rarely incorporated into product labelling, whereas study findings linking drugs to malformations are quickly included in package leaflets (Winterfeld et al., 2014; Crettenand et al., 2018). As patients frequently refer to the package leaflet, while healthcare providers (HCP) predominantly rely on the summary of product characteristics (SmPC), this perpetuates the misconception that all drugs are dangerous during pregnancy (Csajka et al., 2014; Sanz et al., 2001). The result is a widespread distortion of the safety information used by HCP to communicate with their patients.

## 2 Challenges in communicating medication risks during pregnancy

While many medications have not been shown to adversely affect fetal health, certain conditions such as systemic lupus erythematosus, diabetes, or inflammatory bowel disease necessitate careful management during pregnancy. Discontinuing medication may lead to maternal health decline, posing greater risks to the fetus than the potential hazards associated with the medication itself. Thus, optimal disease management during pregnancy is essential for the wellbeing of both mother and child. Medication use is highly prevalent with 27%–93% of women reporting using at least one drug during pregnancy with variations across countries (Mitchell et al., 2011; Lupattelli et al., 2014; Daw et al., 2011). These figures are expected to rise due to higher maternal age at first pregnancy (EUROSTAT, 2021), associated with an increased risk of pre-existing medical conditions and pregnancy complications, and frequent polypharmacy (Fridman et al., 2014; Anand et al., 2023).

The decision to use medication during pregnancy should consider safety data availability, treatment indication, maternal disease severity, and alternative options. In reality, risk perception by both women and HCP are crucial, with studies indicating overestimation of medication-induced malformation risk by both groups (Sanz et al., 2001; Ceulemans et al., 2019). Thus, even when a medication is not expected to adversely affect the health of the fetus, mothers might receive incorrect advice to discontinue essential treatments. This miscommunication poses a serious risk, potentially harming both the mother and child, and, in extreme cases, end in unjustified termination of pregnancy (Schirm et al., 2004; Saha et al., 2015; Einarson et al., 2001a; Polifka and Friedman, 2003; Einarson et al., 2001b). Moreover, regardless of the advice of the HCP, women may choose to discontinue essential medications due to safety concerns or uncertainty about potential adverse effects on the fetus (Watanabe et al., 2021). The overly cautious attitude towards the use of medication during pregnancy is a major issue in communicating medicine, particularly in the field of maternal health and obstetrics. Although the direct impact of communicating distorted information remains relatively unexplored, some evidence of pregnancy disruptive effects has been provided for chronic conditions, such as hypertension, lupus, psychiatric disorders, epilepsy, or inflammatory bowel disease, with observed increase of risk of relapse, hospital admission, and disability in case of avoidance of medication use (Einarson et al., 2001a; Cohen et al., 2004; Cohen et al., 2006; Alcantarilla et al., 2023; Benard-Laribiere et al., 2020; Edey et al., 2014; Leach et al., 2017; Hellwig et al., 2022).

## 3 Role and limitations of current information sources

Whilst there is still insufficient evidence regarding the safety of medicines during pregnancy, where evidence does exist, it should be communicated and factored into the risk assessment. Experts in this domain gather and disseminate this information through specialized up-to-date sources (Table 1). These resources provide comprehensive data, risk classification, therapeutic alternatives, and practical guidance for pregnancy follow-up. In addition to reference books, websites, such as UKTIS (www.uktis.org), Embryotox (in German) (Embryotox, 2018), and Le CRAT (in French) (CRAT, 2018; Elefant et al., 2014), serve as reliable online sources for HCP and are accessible for everyone free of charge. BUMPs (www.medicinesinpregnancy.org) and MotherToBaby (in English) are directed towards parents, addressing the need for accessible information. HCP, and sometimes patients, can also obtain information and individual risk assessments from Teratology Information Services (TIS) (Chambers, 2011). These centers of expertise provide free information and advice, prospectively collect high quality data on reported cases of exposure and outcomes, and pool these data at a network level (e.g., MotherToBaby, European Network of Teratology Information Services–ENTIS (European Network of Teratology Information, 2011)) to improve knowledge on drug safety during pregnancy (Lurie et al., 2021). Despite the availability of various resources, there is currently no specialized information source tailored to address the information needs of both HCP and patients simultaneously.

**TABLE 1:** Information sources for medication use during pregnancy.

Name of source	Description	Hosting country	Target audience	Reference
Online Databases				
Le CRAT	Information on medication use and medication risks during pregnancy, breastfeeding as well as in case of paternal exposure	France	HCPs	Centre de Référence sur les Agents Tératogènes (CRAT). http://www.lecrat.fr
Embryotox	Information on medication use during pregnancy and breastfeeding	Germany	HCPs	Embryotox.https://www.embryotox.de/
Netherlands Pharmacovigilance Centre Lareb	Medication safety information during pregnancy and breastfeeding	Netherlands	HCPs	https://www.lareb.nl/mvm-kennis-zoeken?zoekterm=paracetamol%2Fcodeine&zwangerschap=true&borstvoeding=true
Janusmed	National knowledge database on medication effects during pregnancy and breastfeeding	Sweden	HCPs	https://janusmed.se/fosterpaverkan
UKTIS Monographs	Information on the safety of medications during pregnancy	United Kingdom	HCPs	https://uktis.org/monographs/
Best Use of Medicines in Pregnancy (BUMPs)	Patient information leaflets on medication use during pregnancy	United Kingdom	Patients	bumps - best use of medicine in pregnancy (medicinesinpregnancy.org)
MotherToBaby	Fact sheets on medications and pregnancy	United States	Patients	https://mothertobaby.org/fact-sheets/
Reprotox^®^	Online database on environmental hazards to human reproduction and development	United States	HCPs	REPROTOX^®^ Reproductive Hazard Information. https://reprotox.org/
Shepard’s Catalog of Teratogenic Agents	Information on teratogenic agents including chemicals, food additives, household products, environmental pollutants, pharmaceuticals, and viruses	United States	HCPs	https://www.micromedexsolutions.com/
TERIS Teratogen Information System	Information on the teratogenic effects of drugs and environmental agents	United States	HCPs	https://deohs.washington.edu/teris/
IMI ConcePTION Knowledge Bank	Additional resource of information under development		HCPs and patients	https://www.imi-conception.eu/ F. O’Shaughnessy, B. Cuppers, U.Norby, M. Berlin, P. D. Skold, J. L Richardson. Neurotoxicology and Teratology, 92 (2022):107,108
Reference Books				
Briggs Drugs in Pregnancy and Lactation	Reference guide to fetal and neonatal risk	United States	HCPs	Briggs GG, Forinash AB, Towers CV. Briggs Drugs in Pregnancy and Lactation: A Reference Guide to Fetal and Neonatal Risk. Lippincott Williams and Wilkins; 2021

HCPs: healthcare professionals.

## 4 Discussion

Although currently lacking, a single data source for HCPs and patients, would promote shared decision-making by encouraging information synergy. Shared decision-making is a collaborative process between patients and their HCP, aimed at reaching joint decisions and broad consensus about care based on evidence and informed personal preferences, health beliefs, and values (NICE, 2018; NICE, 2015). This involves ensuring that patients have a good understanding of the risks, benefits, and possible consequences of different options through discussion and information sharing (Stacey et al., 2024). While the benefits of shared-decision making are increasingly recognized, it is not yet routinely practiced in every setting, particularly within the specific field of medication use during pregnancy. A common information tool for patient and HCP aligns with key steps recommended in shared decision-making implementation frameworks (England, 2022). Providing access to evidence-based, language appropriate information for patients and HCPs empowers individuals to make informed decisions and promotes adherence to evidence-based treatment. A common data source serves as a decision support tool tailored to individuals with varying levels of health literacy, facilitating comprehension of available options and associated benefits, harms, consequences, and burdens. It also helps HCPs and patients to make high-value decisions, such as in pregnancy, where decisions about the use of medicines can have a significant impact on the health of both mother and child. Furthermore, a common data source can serve as a training tool for HCPs, specifically in the context of risk communication, facilitating meaningful shared decision-making conversations. Health informatics must also work to address health inequality that exists within most high-income countries. In the UK, for example, compared to white women, there was a more than three-fold increase in maternal mortality in women from black ethnic backgrounds, and mortality was almost doubled in women from Asian ethnic backgrounds. Furthermore, babies born to these women were more likely to be stillborn, or to die in the neonatal period (MBRRACE-UK, 2022). Communication failures including language barriers, and inadequate information or signposting are common themes cited by women from these communities (Khan et al., 2023). Due to socioeconomic factors, these women have more complex medical needs and so the need to deliver accessible information about medicine use and healthcare in general is imperative.

Addressing gaps in medication risk communication during pregnancy requires resources that are reliable, accessible, and tailored to both healthcare providers and patients. Such tools can strengthen shared decision making, promote informed choices, and improve maternal and child health outcomes. Initiatives like MAMA-MEDS in Switzerland illustrate how these approaches can be put into practice.

## Data Availability

The raw data supporting the conclusions of this article will be made available by the authors, without undue reservation.
